# VR Road and Construction Site Safety Conceptual Modeling Based on Hand Gestures

**DOI:** 10.3389/frobt.2019.00015

**Published:** 2019-03-21

**Authors:** Stéphane Côté, Olivier Beaulieu

**Affiliations:** ^1^Bentley Systems, Québec, QC, Canada; ^2^Département d'Informatique et de Génie Logiciel, Université Laval, Québec, QC, Canada

**Keywords:** virtual reality, conceptual road design, construction safety, hand gestures, engineering

## Abstract

Engineering road design and construction site safety design can be lengthy processes, incompatible with situations where only a conceptual design is required, and/or when required by users unfamiliar with such complex pieces of software. In this study, an intuitive road design VR application based on hand gesture was proposed and developed as a proof of concept. Results show the resulting application is easy, natural and fun to use, and could represent a good alternative to more complex design applications.

## Introduction

Road construction is an expensive process that requires careful preparation. Road design is consequently a long process that must consider many factors, including terrain topography and composition, presence of existing buildings and other obstacles, input from local communities, environmental impact, etc., that all have a potential impact on construction cost. In the design process, it is common practice to evaluate various road design “alternatives,” each one being evaluated and compared to the others. CAD software being generally complex, such alternatives take time to prepare, which may delay the overall project. We hypothesized it could be simplified.

Construction of civil infrastructure makes use of large and heavy equipment and material, which exposes construction workers to risks. In the U.S.A., construction-related fatalities represented 21.4% (937) of all the private industry fatalities (4379) in 2015^1^ Consequently, construction site safety is an element of prime importance. Some organizations require builders to request a “safe work permit,” a written record that authorizes specific work to take place. Such permits are used to ensure that potential hazards have been considered with precautions defined and properly applied (Government of Alberta, 2010).

High level conceptual road planning can be done with very simple tools. For instance, kids playing in sandboxes with hand shovels and model trucks do some sort of 3D conceptual design. We hypothesized basic road design could be made as fun and simple as playing in a sandbox, by letting designers do their design in 3D, on the actual site where the road or building will be built, as if they were playing in a sandbox. Similarly, building sites safety features could be designed in a virtual environment that resembles the jobsite. This way, the design process could be made easier, as it would facilitate the exploration of the site from many vantage points including those that are not necessarily accessible on site and therefore increase the chance of foreseeing all potential safety issues, and it could also facilitate the design process by workers not familiar with 3D design tools, as well as the review process by competent authorities.

In this experiment, we proposed 2 VR design tools: one for roads, the other for worksite safety. The tools show the physical environment in the form of a photo realistic reality mesh, and let users add road and/or construction safety elements to the environment. To make the tool easy to use by anyone not familiar with 3D design, we developed a hand gesture recognition feature that lets users add and manipulate elements using their own hands.

While many past research works are related with hand gesture recognition for design, few are specifically related with engineering CAD. A gesture recognition method in a CAD context is presented in Huang and Rai (2014), but only little energy was put on the actual implementation of the CAD design system. Song et al. (2014) describe a “Gaze and Finger” control interface for 3D model manipulation and show its accuracy would be compatible with CAD applications. Dipen et al. (2013) describe a gesture interface for the manipulation of “virtual clay.” Ye et al. (2006) show that a human computer interface that benefits designers during the conceptual design stage.

In this paper, our main contribution is an immersive, photo-realistic, natural, and easy to use system for road and worksite safety conceptual design.

## Materials and Methods

The photo-realistic virtual environment was created by taking photos of a (road or building construction) site, that were assembled into a photorealistic 3D mesh using Bentley Context Capture, a photogrammetry application. The mesh was then loaded in the Unity graphics environment and displayed in VR using the HTC Vive.

**Figure 1:**
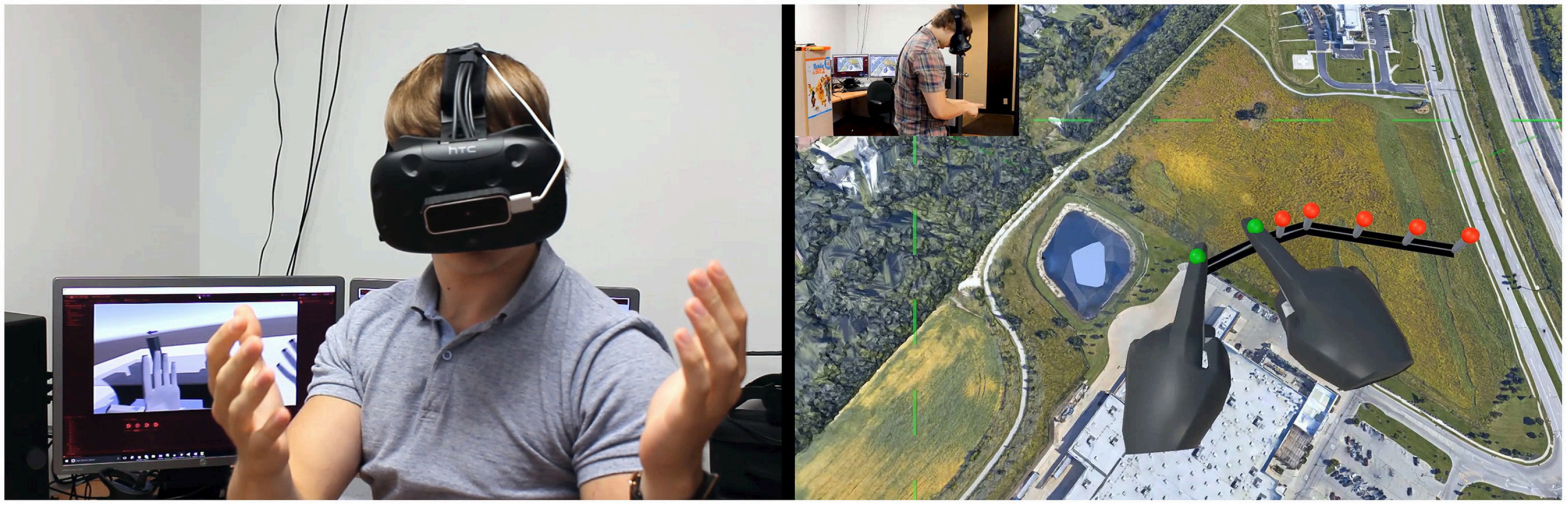
Our setup showing the Leap Motion device affixed onto a HTC Vive VR headset **(Left)**. Virtual hands (on screen) positioned in VR in place of the real user's hands **(Right)**. This figure is being published with the written informed consent of the depicted individual.

Hands were tracked using a Leap Motion device attached onto the Vive, pointing forward (Figure 1), and gestures analyzed using the Leap Motion Orion development kit as well as new code developed in-house. The Orion SDK provides the real-time position and orientation of each phalange, as well as real-time position and a normal vector to each palm, at a rate of 90 fps. Virtual hands were displayed in the virtual world in place of the user's hands (Figure 1).

**Figure 2:**
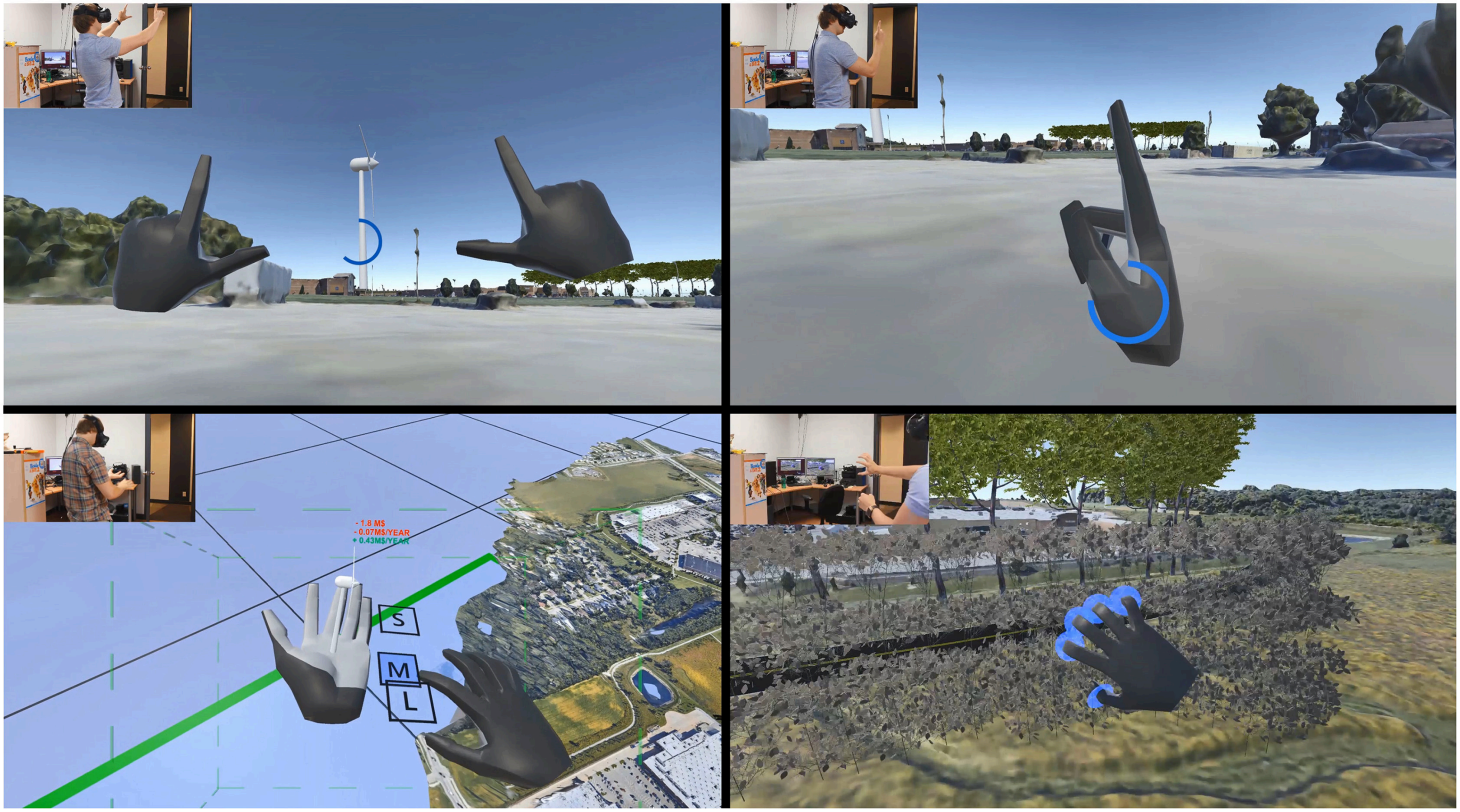
Examples of supported gestures: photo taking **(Top Left)**, teleport **(Top Right)**, pressing menu buttons **(Bottom Left)**, turning dials **(Bottom Right)**. This figure is being published with the written informed consent of the depicted individual.

Two categories of hand gestures were defined. Static gestures included photo taking and teleport (Figure 2, top left and top right) and confirmation (thumbs up), and were recognized by comparing finger relative position and orientation with respect to pre-defined template values and by verifying gesture staticity for a period of about 0.75 s. Dynamic gestures were inspired from real world gestures. They included scaling (closing fists and moving them apart or closer), hand-attached menu display and button selection with visual feedback (touching with fingers, Figure 2, bottom left), manipulating objects (pinch and move), growing trees (turning dial, Figure 2, bottom right), delete (swipe with back of hand), and road drawing and editing. Most of those gestures act the same way as if the user was interacting with the physical world. They were recognized by constantly analyzing the last 15 frames of the hand tracker, and by comparing variations in relative hands position, orientation and fingers relative orientation with pre-defined template values, with some preset tolerance. Only one hand gesture (“Undo”) involved crossing hands, because of a limitation of the Leap Motion Orion SDK dealing with occlusion. Consequently the detection of that gesture, that involved waiving both hands (as to say “no”) involved only palm positions. The gesture was done quickly, so only a few frames had “undefined” positions, which made it possible to recover the movement through the limited 15 frames window.

## Results

Video demos of our prototypes in action are shown in Beaulieu and Côté (2017a,b).

We informally tested the application with 9 colleagues: 4 software developers who had already seen some demos of the project, as well as 3 executives and 2 managers who were not aware of the project. Prior knowledge in road or construction safety design was minimal or none. Comments were mostly positive, and all testers enjoyed using the prototypes.

Hand gestures were almost always recognized correctly when done by the developer of the prototypes. However, recognition rate fell below about 75% when done by other testers. However, some practice was sufficient to raise recognition rate significantly.

Gestures that were closer to real gestures (e.g., picking, dragging, rotation, throwing away) were those that were said to be most intuitive and easy to use by our testers—they picked them up quickly without detailed explanations (they just had to reproduce known gestures they do in the physical world). Other gestures that were mostly symbolic (e.g., taking photos, teleport, undo) were said to be less intuitive, or testers needed more trials before mastering them.

Static gestures (e.g., taking photos, teleport) were mostly well-recognized by our system, and quickly mastered by our testers. Dynamic gestures (e.g., flipping hands to close menus) were not detected as easily. We hypothesize this was due to strict definitions of the gestures, and people not all moving hands the same way: some would do the gestures in a very pronounced way, others not. We also believe that gesture speed may influence recognition rate. Causes for lower recognition rate were not investigated.

The use of photorealistic meshes as context made the design process natural, as testers told us they felt as if they were on site. Live estimated cost analysis (faked, but displayed in real time) made the users aware of road cost as they were designing it, which might shorten the design—cost estimating loop. Animated vehicles (moving in a repeated loop, following a preset realistic path) were added to the construction safety application to make the simulation even more realistic and test potential dangers of machinery moving on site—those vehicles could be seen from various vantage points, from which it was easier to evaluate safety/minimum distances, etc. The use of VR is particularly interesting for detecting potential safety hazards, as it allows one to be onsite and walk in dangerous places without taking any risks. All subjects who tried the applications liked their experience, and said they were very natural and fun to use, some said it was like playing in a sandbox.

## Conclusion

In this experiment, we proposed and developed easy VR tools for the conceptual design of roads and construction site safety based on hand gestures. Our basic results show the applications were natural, easy and fun to use, and could consequently represent a good alternative for more complex 3D engineering design software when only conceptual designs are required, and/or when used by subjects unfamiliar with such complex applications.

Future work should involve improving the robustness of gesture recognition for a variety of users, test with end potential users in the field for feedback, compare design speed and ease of use with existing non-immersive desktop-based conceptual design applications, explore ways to make gestures more robust to various users, and evaluate with users knowledgeable in those fields.

The prototype applications for road and construction safety design feature over 18 different features for navigating, and creating, moving, and deleting elements. That number is vastly inferior to the number of features in CAD applications, but we hypothesize this is nearly sufficient for conceptual design. Missing features would include annotating (for reviewing someone else's design), exporting to CAD, building an itemized list of added elements with positions, capturing video with voice (to let the user explain while showing), and group design sessions.

## Author Contributions

SC conceived the presented idea. OB proposed, developed and tested the computational framework. SC and OB discussed the results and agreed on new features and tests to be done. SC supervised the project, and wrote the manuscript, in consultation with OB.

### Conflict of Interest Statement

The authors declare that the research was conducted in the absence of any commercial or financial relationships that could be construed as a potential conflict of interest.

## Footnotes

^1^United States Department of Labor. https://www.osha.gov/oshstats/commonstats.html
